# A Statewide Hospital-Based Safe Infant Sleep Initiative: Measurement of Parental Knowledge and Behavior

**DOI:** 10.1007/s10900-017-0449-x

**Published:** 2017-11-29

**Authors:** R. L. Walcott, T. C. Salm Ward, J. B. Ingels, N. A. Llewellyn, T. J. Miller, P. S. Corso

**Affiliations:** 1 Department of Health Policy and Management, College of Public Health, University of Georgia, Athens, GA 30602 USA; 2 Helen Bader School of Social Welfare, University of Wisconsin-Milwaukee, Milwaukee, WI 53201 USA; 3 Division of Health Protection and Safety, Georgia Department of Public Health, Atlanta, GA 30303 USA

**Keywords:** Safe sleep education, Sudden infant death syndrome (SIDS), Sleep position, Sleep practices, Infant sleep

## Abstract

Sleep-related infant deaths are a leading cause of infant mortality in Georgia, and these deaths are largely associated with unsafe sleep practices among caregivers. In early 2016, the Georgia Department of Public Health launched the Georgia Safe to Sleep Hospital Initiative, providing hospitals with safe infant sleep information and educational materials to be distributed to families and newborns. This study examined the knowledge and behaviors of a sample of Georgia parents after the implementation of the Hospital Initiative and identified the family characteristics and intervention components most closely associated with the knowledge and practice of safe infant sleep. The primary caretakers of all infants born in Georgia from August to October 2016 were invited to complete a web-based survey 1 month after hospital discharge. The final sample size included 420 parents of newborns, and the primary outcomes assessed included two measures of knowledge and four measures of infant sleep behaviors regarding infant sleep position and location. Most respondents demonstrated knowledge of the correct recommended sleep position (90%) and location (85%). Logistic regression revealed that receipt of information in the hospital was significantly correlated with safe sleep behaviors, and infant sleep habits tended to influence safe sleep practices. Additionally, Medicaid parents receiving bassinets from the hospital were 74% less likely to bed share (OR 0.26; 95% CI 0.007). Implementation of a statewide hospital initiative was associated with high levels of parental knowledge and behavior and may have been successful in reducing the practice of bed sharing among Medicaid parents.

**Electronic supplementary material:**

The online version of this article (10.1007/s10900-017-0449-x) contains supplementary material, which is available to authorized users.

## Introduction

Approximately 3700 sleep-related infant deaths, including sudden infant death syndrome (SIDS), ill-defined deaths, and accidental suffocation or strangulation in bed, occurred in the United States in 2015 [1]. The risk of sleep-related infant death can be reduced if parents follow the safe sleep recommendations from the American Academy of Pediatrics (AAP) regarding the infant’s sleep position (supine), sleep location (in parents’ room on a separate sleep surface), and surrounding environment [1, 2]. Although these recommendations have been widely disseminated since 1992, adherence by parents and caregivers remains low [3, 4]. In Georgia, a statewide survey of maternal attitudes, behavior, and experiences found that only 43.8% of Georgia mothers report always placing their infant on his or her back to sleep, and 48.9% report never sharing a sleep surface with their infants [5]. Sleep-related infant deaths are the third leading cause of infant mortality in Georgia, and of the 158 sleep-related infant deaths that were reviewed in 2014, 82 (51.9%) occurred among infants who were not sleeping on their backs, 95 (60.1%) occurred in an adult bed, and 99 (62.7%) occurred while the infant was sharing a sleep surface with another person (bed sharing) [6].

State-level data have also identified sub-groups at higher risk for engaging in unsafe sleep practices and experiencing sleep-related infant deaths. For example, analyses of the Georgia Pregnancy Risk Assessment Monitory System (PRAMS) data found higher rates of unsafe sleep practices among mothers who: identified as Black or other race or ethnicity (compared to White), were younger, had lower educational attainment, and received Medicaid [5, 7]. While analyzing infant deaths in the state in 2014, the Child Fatality Review Panel found higher proportions of sleep-related infant deaths among infants who were: Black (compared to White), younger (66% of deaths occurred in infants younger than four months), and had younger caregivers [6]. For the years 2010–2013, Medicaid-covered births accounted for 44% of all births in Georgia, but 68% of sleep-related infant deaths occurred among this Medicaid population (Georgia Department of Public Health, unpublished data).

Hospital staff can play a significant role in educating families to reduce the risk of sleep-related infant deaths, as these personnel interact with nearly every new parent in the state. Additionally, research has demonstrated that observing hospital staff modeling safe infant sleep practices reinforces these behaviors for parents [8, 9]. As such, beginning in 2016, Georgia’s Department of Public Health (DPH) launched the Georgia Safe to Sleep Hospital Initiative, a collaboration with birthing hospitals in the state to provide all mothers with safe sleep information and resources as part of a multi-pronged statewide safe infant sleep campaign. This Hospital Initiative was part of a larger statewide collaboration that also included public service announcements and partnerships with social service agencies, the Georgia Children’s Cabinet, the Georgia Bureau of Investigation, and state chapters of professional medical societies. For the Hospital Initiative, DPH provided educational materials to hospitals, including a safe sleep educational flipchart, posters, and handouts for parents. To further reinforce safe sleep practices, DPH also provided educational support materials for hospital staff to distribute to mothers and newborns. All mothers were to receive infant sleep gowns promoting the recommended safe sleep position with “This Side Up” messaging, as well as *Sleep Baby Safe and Snug* board books [10]. Travel bassinets were to be given to all Medicaid and uninsured/self-pay families in order to facilitate compliance with the recommendations for safe sleep location.

Implementation of the Georgia Safe to Sleep Hospital Initiative varied both among and within hospitals during the study period, creating the potential for Georgia parents to receive different “dosages” of the intervention. For example, three hospitals opted out of board book distribution, and only 24 hospitals (30.4%) had fully completed all implementation activities as of March 2017 (T. Miller, et al., 2017, unpublished data). This variation in implementation created a natural experiment with which to examine the effect of the hospital intervention on the safe sleep knowledge and behaviors of primary caregivers. Prior research has examined similar hospital-based interventions [11, 12], with most studies focusing on knowledge and behaviors of hospital staff. A few studies have assessed parental knowledge and behavior one or more months after hospital discharge [13–17], but only two of these studies assessed sleep location as well as sleep position [15, 16]. The purpose of this study was to assess the knowledge and behaviors regarding sleep position and sleep location for a sample of Georgia parents following hospital discharge after birth and to identify the parent characteristics and specific hospital intervention components most closely associated with positive safe sleep knowledge and behavior.

## Methods

The target population for this study included primary caretakers of all infants born in Georgia from August to October 2016. DPH provided the research team with the name, address, and Medicaid status of all mothers giving birth in Georgia during this time period. Postcards containing an invitation to participate in the study were mailed to mothers of newborns in weekly batches 1 month after birth, in order to ensure that the index infant would be at least 1 month old at the time of survey completion. Both the University of Georgia and the Georgia Department of Public Health Institutional Review Boards approved the study protocol.

A web-based survey (with an option to participate by phone) was developed to measure parents’ knowledge and behaviors regarding infant sleep position and location. The survey gathered information including: (1) the safe sleep information and materials received from the birthing hospital and the manner in which hospital education was delivered; (2) self-reported safe sleep knowledge and behaviors regarding infant sleep position and location; (3) self-reported sleep quality and quantity; (4) risk for post-partum depression; and (5) opportunities for parents to provide additional comments and information on their infant sleep practices. Demographic characteristics were also collected. Self-reported race categories included White, Black, or Other (all other races besides “White” or “Black,” all combinations of races, and all who declined to answer). County of birth refers to the county in which the birthing hospital was located and is categorized as rural or non-rural, with rural counties defined by DPH’s Online Analytic Statistical Information System as having a population of < 35,000 [18].

The researchers partnered with DPH to develop survey questions, including validated questions from previously published infant safe sleep studies whenever possible. Knowledge of safe infant sleep recommendations was measured with questions from Hauck, et al. [19], and self-reported infant sleep practices were assessed with items from the PRAMS survey [20]. Sleep quality and quantity were assessed with questions from Dennis & Ross, including how well the infant sleeps (measured ordinally from very poorly to very well), how often the infant cries (measured ordinally from never to very often), and if their infant allowed them to get a reasonable amount of sleep (measured dichotomously) [21]. The Edinburgh Postnatal Depression Scale was incorporated in the survey to identify depressive symptomology, and a cutoff score of 10 (on a scale of 0–30) was designated to identify mothers at risk for depression [22].

Postcards containing information about the study and a link to the online survey were mailed to the addresses of the birth mothers, although any person serving as the infant’s primary caretaker was eligible to complete the survey. A telephone number was also included in case the respondent preferred to complete the survey via phone, along with an incentive of the chance to win a $100 gift card. The web-based survey was offered in both English and Spanish, while the telephone version was offered in English only. Because the researchers were especially interested in Medicaid status as a predictor of knowledge and behavior, the survey links provided on the postcards for Medicaid-covered births were differentiated from those for non-Medicaid-covered births. Approximately 20,500 postcards were mailed.

The primary outcomes assessed included two measures of parental knowledge and four measures of infant sleep behaviors. All six outcomes were scored dichotomously. Table 1 presents each outcome measure with the corresponding survey question, and the final column contains the survey response(s) coded as ‘1’ in dichotomous scoring. The exact survey questions asked and possible responses are reported in Table A (available as a supplement to the online version of this article).

**Table 1:** Study outcomes and associated survey questions/responses

Outcome measure	Survey question	Response coded as 1
Knowledge—infant sleep position	What is the recommended sleep position for healthy babies? [19]	On the back only
Knowledge—infant sleep location	What is recommended about where your new baby should sleep? [19, 27]	In parents’ room, on a separate sleep surface
Behavior—infant sleep position	In which *one* position do you *most often* lay your baby down to sleep now? [20]	On his or her back
Behavior—infant sleeps alone	In the *past 2 weeks*, how often has your new baby slept alone in his or her own crib or bed? [20]	Always; Often
Behavior—room sharing	When your new baby sleeps alone, is his or her crib in the same room where *you* sleep? [20]	Yes
Behavior—bed sharing	How did your new baby *usually* sleep in the *past 2 weeks*? [20]	On a twin or larger mattress or bed

### Data Analysis

Survey responses were analyzed with R Statistics [23]. Descriptive statistics were obtained for maternal and newborn characteristics and compared to available Georgia population estimates using Chi square or t-tests for independence, as appropriate. Logistic regression was used to assess the parent characteristics and intervention components associated with safe sleep knowledge and behavior. The potential predictor variables for each outcome were identified, and stepwise logistic regression models were constructed for each outcome variable using AIC model selection to maximize model fit. For each model, an alpha of 0.05 was used to designate significance of the included predictor variables. A sub-analysis of Medicaid recipients was conducted in order to identify potential predictors within this population of interest.

## Results

Of the 20,500 postcards mailed, 902 (approximately 4%) were returned due to invalid address information in the birth certificate data. Twenty-one respondents did not complete survey items pertaining to safe sleep knowledge and behavior and were dropped from the analysis; outcomes were not imputed. The final sample analyzed included 420 respondents, of whom 128 (30%) were Medicaid recipients. The vast majority of survey respondents (98%) indicated their relation to the infant as “Mother,” and the remaining 2% identified as “Father.” The average age of the index infant at the time of survey completion was 7.8 weeks (range 4–24 weeks). Table 2 displays the demographic characteristics of the survey respondents, as well as selected maternal and newborn characteristics, and compares this sample to available Georgia population estimates. When compared to mothers of all infants born in Georgia in 2016, the respondent sample was less likely to receive Medicaid and more likely to be married, White, more educated, and practice breastfeeding. The geographic distribution of survey respondents throughout the state is comparable to the geographic distribution of all births during this time period.

**Table 2:** Characteristics of survey respondents

Survey respondents (N = 420)		Georgia population	Significance
Characteristic	Average (range)	Average	
Respondent age^a^	30.1 years (16–44)	28.2 years	p < 0.001
Baby age	7.8 weeks (4–24)	–	
Number of children in home	1.9 kids (1–9)	–	

	Number (%)	Percentage	
Medicaid^a^	128 (30.5)	46.7	p < 0.001
Married^a^	309 (73.6)	55.2	p < 0.001
Index infant is only child in home	183 (43.6)	–	
Race^a^			p < 0.001
White	269 (64.0)	56.5	
Black	85 (20.2)	34.4	
Other	66 (15.7)	9.1	
Ethnicity^a^			p = 0.367
Hispanic	51 (12.1)	14.2	
Not Hispanic	369 (87.9)	85.8	
Education^a^			
< High school	21 (5.0)	14.3	p < 0.001
High school/GED	60 (14.3)	29.3	
Some college	76 (18.1)	–	
Tech graduate	25 (6.0)	–	
4 year college graduate	123 (29.3)	–	
Postgraduate degree	115 (27.4)	–	
County of birth^b^			p = 0.947
Non-rural	378 (90.0)	93.3	
Rural	26 (6.2)	6.2	
Home	7 (1.7)	–	
Unknown	9 (2.1)	–	
Postnatal depression risk^c^	54 (12.9)	15.0	p = 0.254
How well does your baby sleep? [21]			
Very poorly	1 (0.2)	–	
Poorly	7 (1.7)	–	
Somewhat well	97 (23.1)	–	
Well	154 (36.7)	–	
Very well	145 (34.5)	–	
My baby lets me get a reasonable amount of sleep [21]	333 (79.3)	–	
How often does your baby cry? [21]			
Never	4 (1.0)	–	
Hardly ever	82 (19.5)	–	
Sometimes	241 (57.4)	–	
Often	66 (15.7)	–	
Very often	9 (2.1)	–	
Breastfeed^d^	386 (91.9)	70.5	p < 0.001

^a^Population data were obtained from DPH for all mothers giving birth in 2016

^b^Population data were obtained from DPH for all births during the study period

^c^Population data were obtained from Salm Ward et al. [34]

^d^Population data were obtained from Salm Ward et al. [24]

### Factors Associated with Knowledge and Behavior

Almost all survey respondents (95%) reported receiving safe sleep information and/or materials in the hospital, with 86% receiving information on infant sleep position (back to sleep) and 73% receiving information on infant sleep location (room sharing, not bed sharing). Seventy-nine percent (79%) of respondents reported receiving at least one of the three Georgia Safe to Sleep Hospital Initiative items: the sleep gown (71%), board book (59%), or bassinet (23%, targeted to Medicaid and uninsured families). There were no significant differences by age, race, or county of birth (rural vs. non-rural) in the distribution of sleep gowns and board books to all respondents or in the distribution of bassinets to Medicaid respondents. Table 3 presents the odds ratios and 95% confidence intervals for the variables selected into each of the six outcome models using AIC model selection, as well as the sample size for each model and the percentage of respondents selecting the outcome choice coded as 1. Table B (available as a supplement to the online version of this article) presents these logistic regression results alongside a full list of all potential predictor variables considered for each model.

**Table 3:** Logistic regression results for full sample, N = 420

Predictor variables^a^	OR (95% CI)
Knowledge: Back to sleep (n = 399^b^) 90% outcome coded as 1^c^	
Parent age	1.9 (1.3, 2.9)
Race	
White	(ref)
Black	0.3 (0.1, 0.6)
Other	0.4 (0.1, 0.95)
Married	0.5 (0.2, 1.1)
Hospital location (non-rural)	3.3 (1.1, 9.2)
Received board book	2.1 (1.0, 4.3)
Received bassinet	0.5 (0.2, 1.2)
Knowledge: Room share (n = 410^b^) 85% outcome coded as 1^c^	
Parent age	0.8 (0.6, 1.0)
Married	1.7 (0.9, 3.2)
Receive info-room share	2.3 (1.3, 4.0)
Behavior: Back to sleep (n = 398^b^) 89% outcome coded as 1^c^	
Race	
White	(ref)
Black	0.3 (0.1, 0.7)
Other	0.9 (0.3, 2.5)
How well does baby sleep	1.7 (1.1, 2.7)
My baby lets me get reasonable sleep	0.4 (0.1, 1.0)
Knowledge (Back to sleep)	3.7 (1.6, 8.5)
Behavior (Baby sleeps in crib)	4.3 (1.8, 9.8)
Behavior: Baby sleeps alone (n = 393^b^) 77% outcome coded as 1^c^	
Married	1.7 (1.0, 2.9)
Receive info-back to sleep	2.3 (1.1, 4.6)
Receive info-room share	1.9 (1.1, 3.2)
How often does baby cry	0.6 (0.4, 0.9)
Knowledge (Back to sleep)	0.5 (0.2, 1.1)
Knowledge (Room share)	0.4 (0.2, 0.9)
Behavior: Room share (n = 374^b^) 76% outcome coded as 1^c^	
Parent age	1.2 (0.9, 1.6)
Index infant is only child in home	0.6 (0.4, 1.1)
Received any information	5.9 (1.1, 37.5)
Receive info-back to sleep	0.2 (0.0, 0.7)
Received board book	1.5 (0.9, 2.5)
Received bassinet	3.6 (1.6, 8.7)
Postnatal depression risk	2.1 (0.9, 5.6)
My baby lets me get reasonable sleep	0.4 (0.1, 0.8)
Knowledge (Back to sleep)	0.4 (0.1, 1.2)
Knowledge (Room share)	4.7 (2.4, 9.1)
Behavior (Baby sleeps in adult bed)	2.1 (1.03, 4.5)
Behavior (Baby sleeps in crib)	3.9 (1.5, 10.4)
Behavior: Baby sleeps in adult bed (n = 381^b^) 24% outcome coded as 1^c^	
Medicaid status	0.6 (0.3, 1.1)
Race	
White	(ref)
Black	3.0 (1.5, 5.9)
Other	5.9 (3.1, 11.5)
Education level	0.7 (0.6, 0.9)
Received any information	0.1 (0.0, 0.4)
How often does baby cry	1.4 (1.0, 2.1)
Breastfeed	2.8 (1.0, 9.4)
Behavior (Back to sleep)	0.5 (0.2, 1.1)

All ordinal variables are coded in increasing order

^a^The table reports odds ratios of all variables selected into each model using AIC model selection; variables were selected to maximize model fit, and selection does not indicate significant association. Full results including all variables considered for each outcome are available in Table B of the online supplement

^b^All 420 respondents did not complete every survey item; therefore each model was constructed with a subset of respondents who completed the survey items included in the model. No responses were imputed

^c^The percentage of respondents whose answer to the survey item of the corresponding outcome was coded as 1

#### Knowledge

Parental age was positively correlated with knowing the recommendation for “back to sleep” (OR 1.9; 95% CI 1.3, 2.9), and parents who gave birth in hospitals in non-rural counties (OR 3.3; 95% CI 1.1, 9.2) were also more likely to know this recommendation. Parents who identified as Black (OR 0.3; 95% CI 0.1, 0.6) or Other (OR 0.4; CI 0.1, 0.95) were less likely than parents who identified as White to know the recommended sleep position. Parents who received information about room sharing in the hospital were more than twice as likely to know this recommendation for safe infant sleep location (OR 2.3; 95% CI 1.3, 4.0).

#### Behavior

There is a high correlation between the knowledge of the recommended infant sleep position and the practice of “back to sleep” (OR 3.7; 95% CI 1.6, 8.5) and the knowledge of the recommended infant sleep location and the practice of room sharing (OR 4.7; 95% CI 2.4, 9.1). Parents who reported receiving information from the hospital on room sharing were significantly more likely to put their infant to sleep alone in his or her crib (OR 1.9; 95% CI 1.1, 3.2), and parents who received a bassinet in the hospital were almost four times more likely to room share than parents who did not receive a bassinet (OR 3.6; 95% CI 1.6, 8.7). However, parents who practiced room sharing were less likely to report that their infant allowed them to get a reasonable amount of sleep (OR 0.4; 95% CI 0.1, 0.8), and babies who cried more often were less likely to be put to sleep alone (OR 0.6; 95% CI 0.04, 0.9). Additionally, parents who reported better infant sleep quality were significantly more likely to practice “back to sleep” (OR 1.7; 95% CI 1.1, 2.7).

Parents who practiced room sharing were significantly more likely to answer in the affirmative for both sleep locations: the crib (OR 3.9; 95% CI 1.5, 10.4) and an adult bed (OR 2.1; 95% CI 1.03, 4.5). Room sharing knowledge and receiving information on “back to sleep” were significantly associated with placing the infant alone to sleep and with room sharing behavior, respectively, though not in the direction expected. Parents who identified as Black were less likely to place their infant on his or her back than parents who identified as White (OR 0.3; 95% CI 0.1, 0.7), and parents who identified as Black (OR 3.0; 95% CI 1.5, 5.9) or Other (OR 5.9; 95% CI 3.1, 11.5) were significantly more likely to place their infant to sleep in an adult bed than parents who identified as White.

#### Medicaid Respondents

A sub-analysis of Medicaid respondents was conducted and is reported in Table C (available as a supplement to the online version of this article). Receiving resources from the hospital was a significant predictor of sleep location for Medicaid respondents, as parents who received the infant sleep gown were nearly five times more likely to room share (OR 4.7; 95% CI 1.3, 17.2) and parents who received bassinets were about 70% less likely to bed share (OR 0.3; 95% CI 0.1, 0.7). Additionally, parents were less likely to place the infant to sleep alone in his or her crib (OR 0.3; 95% CI 0.1, 0.6) and more likely to bed share (OR 3.0; 95% CI 1.1, 8.3) if the index infant was the only child in the home. Bed sharing was also more likely if the infant slept poorly (OR 0.3; 95% CI 0.2, 0.6). As with the full sample, Medicaid parents who identified as Black (OR 6.0; 95% CI 2.0, 20.0) or Other (OR 5.7; 95% CI 1.5, 22.9) were more likely to practice bed sharing than parents who identified as White. Medicaid status, respondent ethnicity, receiving the board book, and postnatal depression risk were not significant predictors of safe infant sleep knowledge or behaviors for the full sample nor for the Medicaid respondents.

## Discussion

A large majority of survey respondents demonstrated knowledge of the recommended sleep position (90%) and the recommended sleep location (85%). These results are comparable to the post-intervention knowledge rate of 94% reported in a study of a community-based safe sleep program [19]. Regarding the practice of the recommended sleep position, 89% of respondents in our sample reported usually placing their newborn on his or her back to sleep. This rate is substantially higher than the 48.9% of respondents who reported usually practicing “back to sleep” in the 2013 Georgia PRAMS survey, which was conducted prior to this Hospital Initiative among caregivers of infants 2–6 months old [24]. This rate is also higher than the 72.6% reported in the National Infant Sleep Position Study (NISP) and the 77.3% reported in a recent national sample [3, 25]. Respondents identifying as White tended to be more likely to practice “back to sleep” and less likely to practice bed sharing than respondents identifying as Black, which is consistent with prior studies [2, 7, 26–28]. In comparison to available state and national-level data on infant sleep knowledge and behaviors, our sample appeared to have a similar level of knowledge of safe sleep recommendations and a higher prevalence of practicing “back to sleep,” indicating that the Georgia Safe to Sleep Hospital Initiative may have been successful in its educational campaign to parents.

Twenty-four percent (24%) of our sample indicated that their infant usually slept in an adult bed, while 32.1% of Georgia PRAMS respondents reported usually sharing a sleep surface with their infant in 2013 [24]. We found that parents who reported that the infant’s crib was in their room (room sharing) were twice as likely to also report that the infant sleeps in an adult bed. These results are in-line with observational studies that have found that infants sleep in multiple locations throughout the night [29–31]. It may be that when room sharing, it is easier to bring the baby to bed for comfort or to preserve parental sleep, both of which are commonly identified as reasons for bed sharing [32].

In a sub-analysis of Medicaid respondents, those who received the bassinet were significantly less likely to report bed sharing when compared to Medicaid respondents who did not receive a bassinet. These results suggest that DPH’s targeted distribution of the bassinet to an identified higher-risk population (Medicaid-covered births) may have been successful in encouraging parents to place their infant on a separate sleep surface and thus reduce the practice of bed sharing. A reduction in bed sharing has the potential to lower the rate of sleep-related infant deaths, as the AAP recommendations suggest that risk of SIDS is cut in half when infants sleep on a separate surface in the parents’ room [1].

The majority of sleep-related infant deaths (68%) from 2010 to 2013 occurred in families receiving Medicaid (Georgia Department of Public Health, unpublished data). While our survey results revealed variation in the rates of safe sleep knowledge and behaviors between Medicaid families and the full sample, we did not find Medicaid status to be a significant predictor of knowledge or behavior in a model containing multiple predictor variables. It is possible that these results suggest an underlying risk factor common but not exclusive to Medicaid recipients that is more closely linked to safe infant sleep knowledge and behavior. The predictors of knowledge and behavior among parents and their linkages to sleep-related infant death should be further explored in future studies.

The survey results also identified barriers to the practice of safe sleep behaviors. In this sample, infants who cried more often were less likely to be placed to sleep alone in a crib, and infants who slept poorly were less likely to be placed “back to sleep.” These findings fit with previous literature, which identified soothing a crying infant as a common reason for bed sharing and perceived infant comfort as a reason for prone sleep position [25, 28]. Additionally, respondents who room shared were less likely to report getting a reasonable amount of sleep; previous literature has found that room sharing is associated with less reported sleep for infants and an increased likelihood to bed share throughout the night [33]. These findings suggest the importance of tailoring safe infant sleep education to mitigate these barriers, such as including information on how to develop healthy sleep hygiene and soothing techniques.

## Limitations

The sample size of the parent survey was modest (N = 420), representing approximately 2% of the total births during the study period. While 2% of the targeted population is low, the final sample size was large enough to run sufficient statistical comparisons. However, the overall sample contained a low proportion of Medicaid recipients and was more educated, White, and married compared to the total population of mothers giving birth in Georgia in 2016. It is possible that the lack of representativeness in our sample contributed to the relatively high percentage of respondents reporting correct knowledge and behaviors of positive infant sleep practices and/or the corresponding low variance in outcomes. Further, self-selection bias exists in our study design, as participation in online survey research requires access to and familiarity with the Internet. Finally, despite the anonymity of the survey, the stigma associated with infant sleep practices may have resulted in response bias.

An additional limitation of this evaluation is its cross-sectional design. Because baseline knowledge and behaviors were not surveyed in a pre-test, it is not possible to assess knowledge or behavior *change* resulting from the Georgia Safe to Sleep Hospital Initiative. The conclusions of the analyses performed in this evaluation are limited to assessing the *association* of the initiative’s components to the outcomes of interest; no outcomes can be directly attributed to the initiative. Further limiting the attribution of study outcomes to the Hospital Initiative is the fact that the initiative was one component of a multi-pronged statewide safe sleep campaign, and parents may have received safe sleep messaging from multiple sources during this time period.

## Conclusions

The core goal of the Georgia Safe to Sleep Hospital Initiative and the broader Georgia Safe Sleep Campaign is to equip all infant caregivers with information and materials promoting safe infant sleep practices in order to reduce the risk factors associated with sleep-related infant death. The results of the parent survey indicate that the vast majority of respondents were aware of the recommendations for safe infant sleep position and location, and receiving information about room sharing in the hospital was strongly correlated with knowledge and behaviors regarding safe infant sleep location. Receiving the bassinet was also significantly correlated with room sharing in the full respondent sample and avoidance of bed sharing among Medicaid respondents. This study can inform the future efforts of other statewide public/private partnerships designed to educate infant caregivers in a hospital setting on safe sleep recommendations in order to reduce the risk factors associated with sleep-related infant death.

## Electronic supplementary material

Below is the link to the electronic supplementary material.

Supplementary material 1 (DOCX 139 KB)
