# Supplementary material for: A Statewide Hospital-Based Safe Infant Sleep Initiative: Measurement of Parental Knowledge and Behavior
Source: J Community Health. 2017 Nov 29;43(3):534–42. doi: 10.1007/s10900-017-0449-x (PMC5919986; doi:10.1007/s10900-017-0449-x)
Supplement: Supplementary file 1 — Supplementary material 1 (DOCX 139 KB) [file 10900_2017_449_MOESM1_ESM.docx]

**Supplement to the online version of the article:**

**“A Statewide Hospital-based Safe Infant Sleep Initiative: Measurement of Parental Knowledge and Behavior”**

Table A contains the variables used in the analysis with their corresponding survey questions and responses. For dichotomous variables, the response coded as ‘1’ is bolded.

Table A: Survey questions and responses for outcome variables and predictor variables

| **Variable** | **Survey question** | **Possible responses**^a^ |
| --- | --- | --- |
| Outcome Variables | | |
| Knowledge – infant sleep position | What is the recommended sleep position for healthy babies?^S1^ | **On the back only**  Side or back is okay  Side only  Side or stomach is okay  Stomach only  I don’t know |
| Knowledge – infant sleep location | What is recommended about where your new baby should sleep?^S1,S2^ | **In parents’ room, on a separate sleep surface**  In parents’ room, in parents’ bed  In own room  Other (open-ended)  I don’t know |
| Behavior – infant sleep position | In which *one* position do you *most often* lay your baby down to sleep now?^S3^ | On his or her side  **On his or her back**  On his or her stomach |
| Behavior – infant sleeps alone | In the *past 2 weeks*, how often has your new baby slept alone in his or her own crib or bed?^S3^ | **Always**  **Often**  Sometimes  Rarely  Never |
| Behavior – room sharing | When your new baby sleeps alone, is his or her crib in the same room where *you* sleep?^S3^ | No  **Yes** |
| Behavior – bed sharing | How did your new baby *usually* sleep in the *past 2 weeks*?^S3^ | In a crib, bassinet, or pack and play  **On a twin or larger mattress or bed**  On a couch, sofa, or armchair  In an infant car seat or swing  In a sleeping sack or wearable blanket  With a blanket  With toys, cushions, or pillows, including nursing pillows  With crib bumper pads (mesh or non-mesh) |
| Predictor variables | | |
| Received any info/materials | Did you receive any information, materials, and/or resources about safe infant sleep in the hospital? | No  **Yes** |
| Received info-back to sleep | At the hospital, did you receive the recommendation: Back to sleep for every sleep? | No  **Yes**  Don’t know/remember |
| Received info-room share | At the hospital, did you receive the recommendation: Room-sharing without bed-sharing? | No  **Yes**  Don’t know/remember |
| Received sleep gown | Did you receive an infant **sleep gown** from the hospital where you had your baby? | No  **Yes** |
| Received board book | Did you receive a *Sleep Baby Safe and Snug* **board book** from the hospital where you had your baby? | No  **Yes** |
| Received bassinet | Did you receive a travel **bassinet** from the hospital where you had your baby? | No  **Yes** |
| Postnatal depression risk | Ten-question Edinburgh Postnatal Depression Scale^S4^ | Cutoff score of 10 to identify mothers at risk for depression |
| How well does baby sleep | How well does your baby sleep?^S5^ | 4-Very well  3-Well  2-Somewhat well  1-Poorly  0-Very poorly |
| My baby lets me get reasonable sleep | Does your baby’s sleep pattern allow you to get a reasonable amount of sleep?^S5^ | No  **Yes** |
| How often does baby cry | In general, how often does your baby cry?^S5^ | 0-Never  1-Hardly ever  2-Sometimes  3-Often  4-Very often |
| Breastfeed | Did you ever breastfeed or pump breast milk to feed your new baby, even for a short period of time?^S5^ | No  **Yes** |
| Behavior (Baby sleeps in crib) | How did your new baby *usually* sleep in the *past 2 weeks*?^S3^ | **In a crib, bassinet, or pack and play**  On a twin or larger mattress or bed  On a couch, sofa, or armchair  In an infant car seat or swing  In a sleeping sack or wearable blanket  With a blanket  With toys, cushions, or pillows, including nursing pillows  With crib bumper pads (mesh or non-mesh) |

^a^ For dichotomous variables, the response coded as ‘1’ is bolded.

Sources:

S1. Hauck FR, Tanabe KO, McMurry T, Moon RY. Evaluation of bedtime basics for babies: a national crib distribution program to reduce the risk of sleep-related sudden infant deaths. *Journal of community health.* 2015;40(3):457-463.

S2. Moon RY, Oden RP, Joyner BL, Ajao TI. Qualitative analysis of beliefs and perceptions about sudden infant death syndrome in African-American mothers: implications for safe sleep recommendations. *The Journal of pediatrics.* 2010;157(1):92-97. e92.

S3. Pregnancy Risk Assessment Monitoring System. Centers for Disease Control and Prevention; 2017. https://www.cdc.gov/prams/questionnaire.htm.

S4. Cox JL, Holden JM, Sagovsky R. Detection of postnatal depression. Development of the 10-item Edinburgh Postnatal Depression Scale. *The British journal of psychiatry.* 1987;150(6):782-786.

S5. Dennis CL, Ross L. Relationships among infant sleep patterns, maternal fatigue, and development of depressive symptomatology. *Birth.* 2005;32(3):187-193.

Table B contains the full logistic regression results for the 420 respondents, while Table C contains the full logistic regression results for the subset of Medicaid respondents (n=128). All predictor variables are listed in the first column, and each tested outcome is listed in the top row.

| Table B: Logistic regression results for full sample, N=420 | | | | | | | | | | | | | |
| --- | --- | --- | --- | --- | --- | --- | --- | --- | --- | --- | --- | --- | --- |
| **Predictor variables** | **Knowledge:**  **Back to sleep**  **OR (95% CI)** | | **Knowledge:**  **Room share**  **OR (95% CI)** | | **Behavior:**  **Back to sleep**  **OR (95% CI)** | | **Behavior:**  **Baby sleeps alone**  **OR (95% CI)** | | **Behavior:**  **Room share**  **OR (95% CI)** | | **Behavior:**  **Baby sleeps in adult bed**  **OR (95% CI)** | |  |
| Medicaid status |  |  |  |  |  |  |  |  |  |  | 0.6 (0.3, 1.1) | |  |
| Parent age | 1.9 (1.3, 2.9) | | 0.8 (0.6, 1.0) | |  |  |  |  | 1.2 (0.9, 1.6) | |  |  |  |
| Race |  |  |  |  |  |  |  |  |  |  |  |  |  |
| White | (ref) | | (ref) | | (ref) | | (ref) | | (ref) | | (ref) | |  |
| Black | 0.3 (0.1, 0.6) | |  |  | 0.3 (0.1, 0.7) | |  |  |  |  | 3.0 (1.5, 5.9) | |  |
| Other | 0.4 (0.1, 0.95) | |  |  | 0.9 (0.3, 2.5) | |  |  |  |  | 5.9 (3.1, 11.5) | |  |
| Ethnicity |  |  |  |  |  |  |  |  |  |  |  |  |  |
| Education level |  |  |  |  |  |  |  |  |  |  | 0.7 (0.6, 0.9) | |  |
| Married | 0.5 (0.2, 1.1) | | 1.7 (0.9, 3.2) | |  |  | 1.7 (1.0, 2.9) | |  |  |  |  |  |
| Index infant is only child in home |  |  |  |  |  |  |  |  | 0.6 (0.4, 1.1) | |  |  |  |
| Hospital location (non-rural) | 3.3 (1.1, 9.2) | |  |  |  |  |  |  |  |  |  |  |  |
| Received any info |  |  |  |  |  |  |  |  | 5.9 (1.1, 37.5) | | 0.1 (0.0, 0.4) | |  |
| Receive info-back to sleep |  |  |  |  |  |  | 2.3 (1.1, 4.6) | | 0.2 (0.0, 0.7) | |  |  |  |
| Receive info-room share |  |  | 2.3 (1.3, 4.0) | |  |  | 1.9 (1.1, 3.2) | |  |  |  |  |  |
| Received sleep gown |  |  |  |  |  |  |  |  |  |  |  |  |  |
| Received board book | 2.1 (1.0, 4.3) | |  |  |  |  |  |  | 1.5 (0.9, 2.5) | |  |  |  |
| Received bassinet | 0.5 (0.2, 1.2) | |  |  |  |  |  |  | 3.6 (1.6, 8.7) | |  |  |  |
| Postnatal depression risk | na | | na | |  |  |  |  | 2.1 (0.9, 5.6) | |  |  |  |
| How well does baby sleep | na | | na | | 1.7 (1.1, 2.7) | |  |  |  |  |  |  |  |
| My baby lets me get reasonable sleep | na | | na | | 0.4 (0.1, 1.0) | |  |  | 0.4 (0.1, 0.8) | |  |  |  |
| How often does baby cry | na | | na | |  |  | 0.6 (0.4, 0.9) | |  |  | 1.4 (1.0, 2.1) | |  |
| Breastfeed | na | | na | |  |  |  |  |  |  | 2.8 (1.0, 9.4) | |  |
| Knowledge (Back to sleep) | na | | na | | 3.7 (1.6, 8.5) | | 0.5 (0.2, 1.1) | | 0.4 (0.1, 1.2) | |  |  |  |
| Knowledge (Room share) | na | | na | |  |  | 0.4 (0.2, 0.9) | | 4.7 (2.4, 9.1) | |  |  |  |
| Behavior (Back to sleep) | na | | na | | na | |  |  |  |  | 0.5 (0.2, 1.1) | |  |
| Behavior (Baby sleeps alone) | na | | na | |  |  | na | | na | |  |  |  |
| Behavior (Room share) | na | | na | | na | | na | | na | |  |  |  |
| Behavior (Baby sleeps in adult bed) | na | | na | |  |  |  |  | 2.1 (1.03, 4.5) | | na | |  |
| Behavior (Baby sleeps in crib) | na | | na | | 4.3 (1.8, 9.8) | |  |  | 3.9 (1.5, 10.4) | | na | |  |
| Sample size for model^a^ | n=399 | | n=410 | | n=398 | | n=393 | | n=374 | | n=381 | |  |
| Percent outcome coded as 1^b^ | 90% | | 85% | | 89% | | 77% | | 76% | | 24% | |  |
| NOTES: All predictor variables are listed in the first column. All tested outcomes are listed in the top row. The table reports odds ratios of all variables selected into each model using AIC model selection; variables were selected to maximize model fit, and selection does not indicate significant association. Variables that are blank under an outcome were not selected into the model, and na signifies that the variable was not considered as a predictor for the model. All ordinal variables are coded in increasing order.  ^a^ All 420 respondents did not complete every survey item; therefore each model was constructed with a subset of respondents who completed the survey items included in the model. No responses were imputed.  ^b^ This row indicates the percentage of respondents whose answer to the survey item of the corresponding outcome was coded as 1. | | | | | | | | | | | | |  |

| Table C: Logistic regression results for Medicaid sample, N=128 | | | | | | | | | | | | |
| --- | --- | --- | --- | --- | --- | --- | --- | --- | --- | --- | --- | --- |
| **Predictor variables** | **Knowledge:**  **Back to sleep**  **OR (95% CI)** | | **Knowledge:**  **Room share**  **OR (95% CI)** | | **Behavior:**  **Back to sleep**  **OR (95% CI)** | | **Behavior:**  **Baby sleeps alone**  **OR (95% CI)** | | **Behavior:**  **Room share**  **OR (95% CI)** | | **Behavior:**  **Baby sleeps in adult bed**  **OR (95% CI)** | |
| Parent age | 1.4 (0.9, 2.3) | |  | | 3.5 (1.5, 9.8) | |  |  |  | |  |  |
| Race |  |  |  |  |  |  |  |  |  |  |  |  |
| White | (ref) | | (ref) | | (ref) | | (ref) | | (ref) | | (ref) | |
| Black |  | |  |  | 0.1 (0.0, 0.6) | | 0.3 (0.1, 0.9) | |  |  | 6.0 (2.0, 20.0) | |
| Other |  | |  |  | 0.7 (0.1, 4.6) | | 0.3 (0.1, 1.1) | |  |  | 5.7 (1.5, 22.9) | |
| Ethnicity |  |  |  |  |  |  |  |  |  |  |  |  |
| Education level |  |  | 0.8 (0.5, 1.1) | | 0.5 (0.3, 0.9) | |  |  | 0.6 (0.3, 0.9) | |  | |
| Married |  | | 3.0 (1.0, 10.9) | | 0.2 (0.0, 0.9) | |  | |  |  |  |  |
| Index infant is only child in home |  |  |  |  |  |  | 0.3 (0.1, 0.6) | |  | | 3.0 (1.1, 8.3) | |
| Hospital location (non-rural) |  | |  |  |  |  |  |  |  |  |  |  |
| Received any info |  |  |  |  |  |  | 6.6 (1.0, 58.4) | | 44.7 (4.5, 1104.8) | |  | |
| Receive info-back to sleep | 2.7 (0.9, 7.8) | |  |  |  |  |  | |  | |  |  |
| Receive info-room share |  |  | 7.0 (2.5, 21.4) | |  |  |  | |  |  |  |  |
| Received sleep gown |  |  |  |  | 3.6 (0.8, 16.4) | |  |  | 4.7 (1.3, 17.2) | |  |  |
| Received board book |  | |  |  |  |  |  |  |  | |  |  |
| Received bassinet |  | |  |  |  |  | 2.0 (0.8, 5.1) | |  | | 0.3 (0.1, 0.7) | |
| Postnatal depression risk | na | | na | |  |  |  |  | 0.3 (0.1, 1.2) | |  |  |
| How well does baby sleep | na | | na | | 3.0 (1.3, 7.9) | |  |  |  |  | 0.3 (0.2, 0.6) | |
| My baby lets me get reasonable sleep | na | | na | |  | |  |  |  | |  |  |
| How often does baby cry | na | | na | |  |  |  | |  |  |  | |
| Breastfeed | na | | na | | 8.2 (1.2, 60.5) | |  |  |  |  |  | |
| Knowledge (Back to sleep) | na | | na | | 4.9 (1.2, 21.3) | |  | | 0.0 (NA) | |  |  |
| Knowledge (Room share) | na | | na | |  |  |  | |  | |  |  |
| Behavior (Back to sleep) | na | | na | | na | |  |  |  |  |  | |
| Behavior (Baby sleeps alone) | na | | na | |  |  | na | | na | |  |  |
| Behavior (Room share) | na | | na | | na | | na | | na | |  |  |
| Behavior (Baby sleeps in adult bed) | na | | na | |  |  |  |  |  | | na | |
| Behavior (Baby sleeps in crib) | na | | na | |  | |  |  |  | | na | |
| Sample size for model^a^ | n=128 | | n=127 | | n=121 | | n=128 | | n=123 | | n=120 | |
| Percent outcome coded as 1^b^ | 85% | | 84% | | 85% | | 75% | | 86% | | 26% | |
| NOTES: All predictor variables are listed in the first column. All tested outcomes are listed in the top row. The table reports odds ratios of all variables selected into each model using AIC model selection; variables were selected to maximize model fit, and selection does not indicate significant association. Variables that are blank under an outcome were not selected into the model, and na signifies that the variable was not considered as a predictor for the model. All ordinal variables are coded in increasing order.  ^a^ All 420 respondents did not complete every survey item; therefore each model was constructed with a subset of respondents who completed the survey items included in the model. No responses were imputed.  ^b^ This row indicates the percentage of respondents whose answer to the survey item of the corresponding outcome was coded as 1. | | | | | | | | | | | | |
